# Classification of Biomedical Texts for Cardiovascular Diseases with Deep Neural Network Using a Weighted Feature Representation Method

**DOI:** 10.3390/healthcare8040392

**Published:** 2020-10-10

**Authors:** Nizar Ahmed, Fatih Dilmaç, Adil Alpkocak

**Affiliations:** Department of Computer Engineering, Dokuz Eylul University, Tinaztepe Kampusu, 35160 Izmir, Turkey; nizar.ahmed@ceng.deu.edu.tr (N.A.); fatih.dilmac@ceng.deu.edu.tr (F.D.)

**Keywords:** biomedical text classification, multiclass classification, cardiovascular diseases, deep neural network, feature representation, bidirectional long short-term memory, BLSTM

## Abstract

This study aims to improve the performance of multiclass classification of biomedical texts for cardiovascular diseases by combining two different feature representation methods, i.e., bag-of-words (BoW) and word embeddings (WE). To hybridize the two feature representations, we investigated a set of possible statistical weighting schemes to combine with each element of WE vectors, which were term frequency (TF), inverse document frequency (IDF) and class probability (CP) methods. Thus, we built a multiclass classification model using a bidirectional long short-term memory (BLSTM) with deep neural networks for all investigated operations of feature vector combinations. We used MIMIC III and the PubMed dataset for the developing language model. To evaluate the performance of our weighted feature representation approaches, we conducted a set of experiments for examining multiclass classification performance with the deep neural network model and other state-of-the-art machine learning (ML) approaches. In all experiments, we used the OHSUMED-400 dataset, which includes PubMed abstracts related with specifically one class over 23 cardiovascular disease categories. Afterwards, we presented the results obtained from experiments and provided a comparison with related research in the literature. The results of the experiment showed that our BLSTM model with the weighting techniques outperformed the baseline and other machine learning approaches in terms of validation accuracy. Finally, our model outperformed the scores of related studies in the literature. This study shows that weighted feature representation improves the performance of the multiclass classification.

## 1. Introduction

The task of text classification (TC) has evolved during the last decade to become one of the most interesting fields in machine learning. Assigning classes to unseen data by discovering patterns that each class can share is the most motivating challenge in TC. However, there is a prior stage that a classification model must go through before categorizing the data, i.e., feature representation. Feature representation is the step of converting the text data into an appropriate form, such as floating-point numerical vectors, recognizable by the classifier [1]. The more efficient the feature representation method used, the better the ability of the classifier to discover patterns amongst the data [1,2].

Bag of words (BoW) and word embeddings (WE) are the two commonly used feature representation techniques in TC. These two techniques are powerful in classification systems, but their working mechanism differs from one to another. Bag of words (BoW) is a technique that represents the whole-body text, whether documents or sentences, as a list of words. These words are stored in a matrix to be calculated later, regardless of their sentential form and ignoring their syntax, grammar and the semantic relations between them. Term frequency (TF) and Term frequency-inverse document frequency (TF☓IDF) are examples of BoW methods. On the other hand, word embedding (WE) techniques have the ability to discover syntax and context relations between words [1,2]. Both techniques have important advantages in representing data features, but it may be more beneficial to create a powerful weighted feature representation model by combining both of them. In this manner, one may take both techniques’ advantages into account while preparing the classification system. For this reason, there are many classification systems that can use advanced weighted feature representation, such as multiclass and multilabel classification systems.

Some may confuse multiclass and multilabel systems, perhaps thinking them to be the same. But these systems are completely different. A multiclass classification system aims to classify documents into a single class over multiple (i.e., more than two) classes [3]. On the other hand, multilabel systems assign one or more classes to a particular document [4]. Both classification systems suffer from common problems such as class imbalance, dealing with a large number of classes and label dependency. Therefore, both classification systems try to highlight those issues and propose proper solutions to solve them.

Class imbalance is a serious issue, especially if one is dealing with a large number of classes. The major classes, that are related to many documents, will dominate the classification and that may result in false positives or negatives. Meanwhile, for a large number of classes, this issue plays another important role in the classification process. The more labels involved, the more complicated the classification process. Alternatively, the label dependency problem can be useful for multilabel models but harmful for multiclass systems. In multilabel systems, the more classes there are dependent on each other, the more efficient it is to assign multiple classes to a particular document [4], whereas, in a multiclass problem, each document must preserve unique features of a particular class rather than share them with other classes. It is hard to ensure label independence in that case, which leads to a negative impact on the performance side of the classification system [3]. Eventually, all these problems may be partially or completely solvable by enhancing feature representation methods.

The main contribution of this paper is to provide a weighted feature representation method for biomedical text classification, and implementation in a case study of cardiovascular diseases. Our research primarily focuses on combining two different feature representation techniques, such as WE and BoW, to enhance the performance of a biomedical multiclass text classification system. Firstly, we created a language model for feature representation using two popular biomedical datasets, namely PubMed and MIMIC-III, in training. Then, we proposed a series of weighting approaches to combine the two feature representations. Afterwards, we evaluated them with a series of experiments using different machine learning algorithms and we showed that our weighted feature representation method worked well in multiclass classification of biomedical texts.

The rest of this paper is organized as follows. Section 2 provides some important studies regarding the enhancement of WE systems. Section 3 presents our methodology, which begins from the collection of the datasets, the creation of the feature representation techniques, the creation of the hybrid models and, finally, testing on a bidirectional LSTM deep learning model. Section 4 presents our experimental set-up process, shows our results and presents a discussion on them. Finally, Section 5 concludes the paper and presents a summary of our system, our findings and gives a projection on this problem for possible future work.

## 2. Related Work

Several studies in the literature have addressed valuable works on word embeddings for biomedical text classification purposes. For example, the Sentence2Vec word embedding system has been used to build features of vectors [5] This technique works as a sentence-based WE (i.e., each vector represents a whole sentence instead of a word). Later, the word embedding system was tested on a multiclass classification problem that depends on classifying the multiclass hallmarks of the cancer corpus [5]. Another study [4] dealt with the creation of a biomedical WE system in which an attempt was made to construct a biomedical WE system by combining several benchmark WE systems, namely FastText, Word2Vec and Glove. It was considered that benefits of these WE systems could be combined by combining the vectors that resulted from each. The combined WE system was used to classify Spanish clinical electronic health records (HER) which were annotated with ICD-10 codes [4]. On the other hand, Xang et al. [6] proposed a biomedical WE system that depends on sentence encoders to create the vectors, which was used as a feature representation method on their multilabel classification model. After that, they built a convolutional residual neural network model to classify EHR cohort collected from patients at Duke Hospital. Additionally, they used a Boltzmann machine to capture and detect label dependency. Generally, all of the previously mentioned studies proved that, by using a domain-based WE in the biomedical field, it is possible to make a difference in performance rather than using a nonmedical WE. Hence, there is plenty of research that aims to develop and enhance the current WE systems.

Nowadays, enhancing work via WE systems is taking place in the biomedical and nonbiomedical machine learning (ML) society. Improvements have resulted from exploring different datasets [2,5,7,8], different WE systems [9,10,11,12] or by merging different feature representation techniques [13,14]. Since enhancing WE by combining several feature representation techniques is within the scope of this study, we will only focus on addressing its related research here. The combination of several feature representation methods can be done in two ways: either within WE systems, or by combining two or more different feature representation techniques. For example, Pagliardini et al. [15] worked on creating a WE system in sentence-based form and called it Sent2Vec. The combination process was done by taking the average of the vectors of words that reside within the context of a particular sentence. On the other hand, Le and Mikolov [16] built a document-based WE system that mapped one document into a single vector. All the vectors of the words in a particular document were averaged or concatenated to represent one unique vector. However, there are several studies that have combined two or more different feature representation techniques such as WE and BoW. Enríquez et al. [13] constructed a sentiment classification system depending on combining WE and BoW. The combination method depended on a simple voting system which generates a confidence value of both feature representation techniques. Since these values are between zero and one, they calculated an average of the values from both techniques. On the other hand Hu et al. [17] created a recommendation system that retrieves similar bug reports. Their system aimed to employ four different vector representations, or similarity scores, from four different techniques. The first score considered the BoW method and the second one considered WE. The third similarity score was created by bug product and component, which focused more on the structural relationship between two bugs. And finally, the fourth similarity score depended on the latent relationship between two bugs at a document-level. As a result, the combination technique used considered the final score = (score1 + score2 + score4) × score3. At that point, the system recommended the most similar k bug reports to a given query bug. Schmidt [18] tried to improve the work on word embedding systems by combining them with some TF-IDF weighting techniques. They performed several weighting techniques, namely term frequency (TF), inverse document frequency (IDF) and smooth inverse frequency, accompanied with a subsampling function used in a Word2vec model. However, the created word embedding vectors were implemented at a document level which aggregated all the relevant word vectors. Then, they calculated the weighted sum of each weighting technique for a particular word with its related word embedding vector. According to their results, the IDF weighting technique outperformed the others in terms of ROC and AUC performance metrics.

Liu et al. [19] represented another weighted approach that depended on BoW and WE. The combination technique started with calculating the weights of each term in the corpus. Their weighting approach depended on two common BoW methods, namely TF-IDF and class probability. Class probability is the frequency of each term within a particular class. They assumed that their model could recognize the importance and/or the uniqueness of terms in a specific class domain by adding class probability information to TF-IDF weights. After that, they accumulated the resulting weights with each term vector using a simple multiplication operation. Zhou et al. [20] performed another valuable study that used hybridization of three NLP approaches, namely BoW, topic modeling and word embeddings. First, they calculated TF-IDF scores of each term within a particular document, then they used an LDA topic modeling approach to represent topics with terms. After that, they extracted word embedding vectors for all the terms in the corpus. They tried to combine the former mentioned approaches in two ways. The first one combined TF-IDF scores of a particular word with its related word vector, then the updated vectors were joined at the end with LDA scores, whereas the second weighting approach combined TF-IDF and WE as a first step. They combined LDA scores and WE vectors as a second step and, finally, combined the results of both weighting approaches. In general, all the weighted feature representation techniques mentioned above proved their efficiency to enhance the performance of some ML problems. But there are some ML challenges that may need such progress to improve their overall performance, such as multiclass and multilabel classification systems.

The multiclass classification problem is a hot topic in ML, particularly if it involves a biomedical domain. Moreover, researchers have tried their best to solve many related problems, such as label imbalance, label dependency and the low existence of biomedical multiclass datasets. Sinoara et al. [2] created two customized nonbiomedical WE systems called NASAI and Bable2vec embeddings. They evaluated their WE model on a multiclass biomedical dataset called OHSUMED-400. Additionally, this dataset was annotated with 23 cardiovascular diseases and gave lower classification accuracy, i.e., a 37% micro average F1 score. In their opinion, the nonbiomedical WE systems and the complications in the dataset were the reasons behind this low score. Another study tried to solve the high data imbalance problem [21] in which a clinical multiclass dataset consisting of 139 symptoms from 3 different syndromes was considered. A multiple asymmetric partial least squares classifier (MAPLSC) was proposed to solve the data imbalance issue. They adjusted the asymmetric partial least squares classifier (APLSC), which is a binary classifier, to make it deal with a multiclass problem. APLSC is a pairwise coupling strategy for merging the probability of all the one-vs-one binary classifier outputs, leading to estimations of the posterior probabilities for all candidate labels. Finally, Lei et al. [3] addressed research that proposed a solution of data dependency and its negative impact on the multiclass problem. Since some multiclass problems suffer from the large number of labels, they created two approaches that identified data-dependent error bounds. Basically, one of them depended on Gaussian complexity and the other on Rademacher complexity. After that, they established an upper and lower boundary to control the retrieved classes. Both of these techniques could identify correlation among label-wise components. Hence, data-dependent bounds showed an improved dependency on the number of classes. The work is still moving towards improving the performance of multiclass classification systems, even though it is hard to accomplish.

## 3. Method

In this section we present a detailed description about each approach we proposed in this study. Our method first describes how we created the weighting feature representation system starting from the collection of the dataset to calculating the word-embedding vectors from a part of the collected dataset. It also presents the details of calculating term frequency (TF), inverse document frequency (IDF) and class probability (CP) weights for each word and combinations of them. After that we describe how we created the multiclassification model to examine our weighting approach.

### 3.1. Datasets and Data Preparation

We used three types of biomedical datasets which are available in the literature. We used two of them, PubMed and MIMIC-III, for developing the model where our weighting feature representation approach was used. The third one is the OHSUMED dataset, which is a multiclass dataset, and we used it to evaluate our proposed weighting feature representation system. Below, we provide brief information about each of the three datasets:

1. PubMed is one of the most familiar biomedical databases that facilitate access to the MEDLINE biomedical database which includes research papers and is widely used for keeping track of citations. The PubMed database is 170GB (according to statistics from 27 January 2020) in size, and includes approximately 32 million biomedical articles, abstracts, bibliometrics, citations and related data which are stored and updated regularly in our local repository. According to many researchers in biomedical studies looking for reliable evidence-based biomedical resources, PUBMED is considered a benchmark for any biomedical applications that use machine learning models [5,22]. Due to some limitations of our computer environment, we were limited to work with two million Pubmed abstracts rather than the whole collection. Table 1 shows statistics about the collection we used.
2. MIMIC III stands for Biomedical Information Mart for Intensive Care, and contains more than 40,000 patient records from intensive care units of the Beth Israel Deaconess Biomedical Center between 2001 and 2012 [23]. Furthermore, the dataset is related to 53,423 hospital admissions of adults and 7870 records related to neonates. Consequently, more than 20 million clinical summaries are reported in this database. In our case, we used all the existing 20 million patients’ textual clinical summaries. Table 1 shows a brief description of this dataset.
3. OHSUMED-400 is a subset of medical abstracts extracted from the MEDLINE database [24]. This dataset was launched and organized by William Hersh and colleagues at the Oregon Health Science University [25]. Each document of this set is attached with specifically one class over 23 cardiovascular disease categories. The total number of the collected dataset is 13,929 documents. Since the collected documents are highly unbalanced, we under-sampled the data to be more balanced and convenient for the multiclassification system. Accordingly, each class of the 23 cardiovascular diseases related to 400 documents. Eventually, we used 9200 abstracts in total, 70% as training and 30% as testing data. Table 1 summarizes all the necessary information about the OHSUMED-400 dataset.

### 3.2. Data Preprocessing

In this step we used some data preprocessing techniques and libraries to clean up our datasets from undesired characters and punctuation marks. We used the NLTK Python libraries to identify and clean the dataset. For that purpose, we applied the following operations:

- Removed punctuation marks.
- Removed numbers.
- Normalized all characters into lowercase.
- Word tokenization.

### 3.3. Weighted Feature Representation

This step consists mainly of three sub-steps. The first one describes how we calculated the TF, IDF and CP values of each word in the corpus. The second step represents how we created our word embedding vectors. The third step shows how we combined the former two methods as one improved features representation.

To clarify the definition, let us assume that we have a dataset *D*, which includes *m* text documents to classify, *D* = {*d*_1_, *d*_2_, *d*_3_, …, *d_m_*} where an arbitrary document *d_i_* is represented by a set of terms. *d_i_* = {*t*_1_, *t*_2_, *t*_3_, …, *t_n_*}, and each term *t_i_* can be represented as one weighting score such as term frequency score (TF), inverse document frequency (IDF) or class probability (CP) score. In our case *w_j_* represents one of the former mentioned weighting techniques of the term *t_j_* in the document *d_i_* = <*w*_1_, *w*_2_, *w*_3_, …, *w_n_*>.

The whole set of terms appearing in dataset *D*, constitutes a vocabulary of text documents, *V* = {*t*_1_, *t*_2_, *t*_3_, …, *t_n_*} where an arbitrary term *t_i_* is represented by a feature set of *F_i_*, which contains two different features (i.e., weighted value and word embedding vectors) *F_i_* = {*s_i_*, *we_i_*} where *s_i_* and *we_i_* are the weighting score and word embedding vectors of *i*th term, respectively.

#### 3.3.1. Calculation of Weighting Techniques

For this step, we calculated all the weighting scores for each distinct word in the evaluation OHSUMED-400 dataset. We depended on Formulas (1) and (2) for calculating the TF and IDF [18] scores for all the vocabularies in OHSUMED-400 dataset. Formula (3) was used to calculate Class Probability CP [19].

$TF_{t_{j}}$ is a function that returns the term frequency normalized by number of terms in document *d_i_*. This weighting technique shows how a particular term *t_i_* is important in accordance with its co-occurrence in document *d_i_*. Thus, we want to investigate how this technique improves the classification performance when it is accompanied with the word embedding vector.

(1) $$TF_{t_{j}}=\frac{f_{t_{j},d_{i}}}{\left|d_{i}\right|}$$

where *f_tj,di_* represents the number of times the term *t_j_* appears in a document *d_i_*, and |*d_i_*| shows the size of the document in the number of terms.

$IDF_{t_{j}}$ relatively represents the inverse document frequency of term *t_j_* and can be defined as in Equation (2).

(2) $$IDF_{t_{j}}=\;log\frac{N}{m}$$

where *N* represents the number of documents containing the term *t_j_* and *m* represents the total number of documents in the dataset *D*. The IDF weighting technique represents how a particular term *t_i_* is rarely or more frequently occurs in the corpus. For example, more frequent words, such as stop words, will have lower IDF weights than rare words. Accordingly, this technique determines the importance of rare and domain-based words, such as medical terms, in our corpus.

Class probability P(*t_j_*|*C_k_*) or CP*_tj,Ck_,* on the other hand, is the conditional probability that shows how many times the term *t_j_* occurs when the class *C_k_* is appearing. CP demonstrates the importance of a specific term *t_i_* taking into consideration class information. For example, if a term *t_i_* appears multiple times in documents that are annotated as class *A*, this means the term is highly related to that class.

(3) $$CP_{t_{j,Ck}}=\;\frac{t_{j}\cap c_{k}}{P\left(c_{k}\right)}$$

where P(*C_k_*) represents the probability of class *C_k_*. As a result, each term *t_j_* within document *d_i_* the dataset is represented by three weighted scores:

(4) $$sj=\overset{m}{\underset{d=0}{\sum }}s_{j}=\left(TF_{t_{j}},\;IDF_{t_{j}},CP_{t_{j}}\right)$$

Accordingly, after applying the previous equations to the OHSUMED-400 dataset, each word in the vocabulary is now related to the three weighted values *s_j_*. For example, assuming that the document *d_2_* contains only three words, *V* = {*t_1_*^leukemia^, *t_2_*^blood^, *t_3_*^antiglobulin^}, *s_j_* score values are as follows:

*s*_1_^leukemia^ = (TF^leukemia^ = 0.75, IDF^leukemia^ = 0.66, CP^leukemıa^ = 0.99)

s_2_^blood^ = (TF^blood^ = 0.18, IDF^blood^ = 0.36, CP^blood^ = 0.56)

s_3_^antiglobulin^ = (TF ^antiglobulin^ = 0.59, IDF ^antiglobulin^ = 0.98, CP ^antiglobulin^ = 0.43)

#### 3.3.2. Creation of Word Embedding (WE) Vectors

To create a word embedding vector, we used two datasets, Pubmed and MIMIC III, including four million documents in total. We applied the FastText WE library [11] and calculated a 100-dimensional vector space for each word in the vocabulary. FastText produces a powerful model that has solved many natural language issues such as word morphology and out-of-vocabulary word problems [1]. For that reason, we used the Gensim Python library to construct word vectors from the four million documents. As a result, we had a vocabulary *V*, which included all distinct terms in the dataset D, *V* = {*t*_1_, *t*_2_, *t*_3_, …, *t_n_*} where each term is represented with a 100-dimensional WE vector.

For simplicity, assuming that we have only three words in *V*, after running FastText library to extract the feature, the resulting 100-dimensional word embedding vector, *we,* for each term in *V* is defined as follows:

*we*_1_^leukemia^ = < −3.215408, −4.233652, …, 0.2386677> *we*_2_^blood^ = <−6.8077517, 7.012626, …, 3.39976> *we*_3_^antiglobulin^ = <1.4542218, 0.642345, … , −3.6465673> (5)

The values of *we* vectors are the real values taken from our training models, for each respective term.

#### 3.3.3. Weighted Word Embedding Vectors

In this section, we present how to combine WE vectors and the weighting scores to update the structure of the recent or the original WE vector form. This means for every term *t_j_* in the vocabulary *V*, we applied a simple weighting factor to combine WE vectors and the three weighting scores. 

Let *t_j_* represent a particular word in a vocabulary *V*. Then for an arbitrary term *t_j_* related with three weighting scores in (*s_j_*) and each item of its related WE vector (*we_j_*), the weighted multiplication can be performed as follows, and results with the three weighted feature vectors, *HTF_j_, HIDF_j_* and *HCP_j_*. of the three weighting techniques TF, IDF and CP, respectively.

Term frequency (TF) is a weighting scheme. It simply multiplies the TF score of each term *t_j_* with every item in its *we_j_* vector.

(6) $$HTF_{j}=\overset{m}{\underset{l=0}{\sum }}TF_{t_{j}}\times we_{j,l}$$

For example:

*H*_leukemia_ = *TF*_leukemia_ × *we*_leukemia,l_

*H*_leukemia_ = (0.1108 × (−3.215408), 0.1108 × (−4.233652), …, 0.1108 × (0.2386677))

*H*_leukemia_ = = (−0.356108, −0.468879, …, 0.026432).

Similarly, IDF and class probability (CP) scores can be applied as weights as follows:

(7) $$HIDF_{j}=\overset{m}{\underset{l=0}{\sum }}IDF_{t_{j}}\times we_{j,l}$$

(8) $$HCP_{j}=\overset{m}{\underset{l=0}{\sum }}CP_{t_{j}}\times we_{j,l}$$

**Figure 1:**
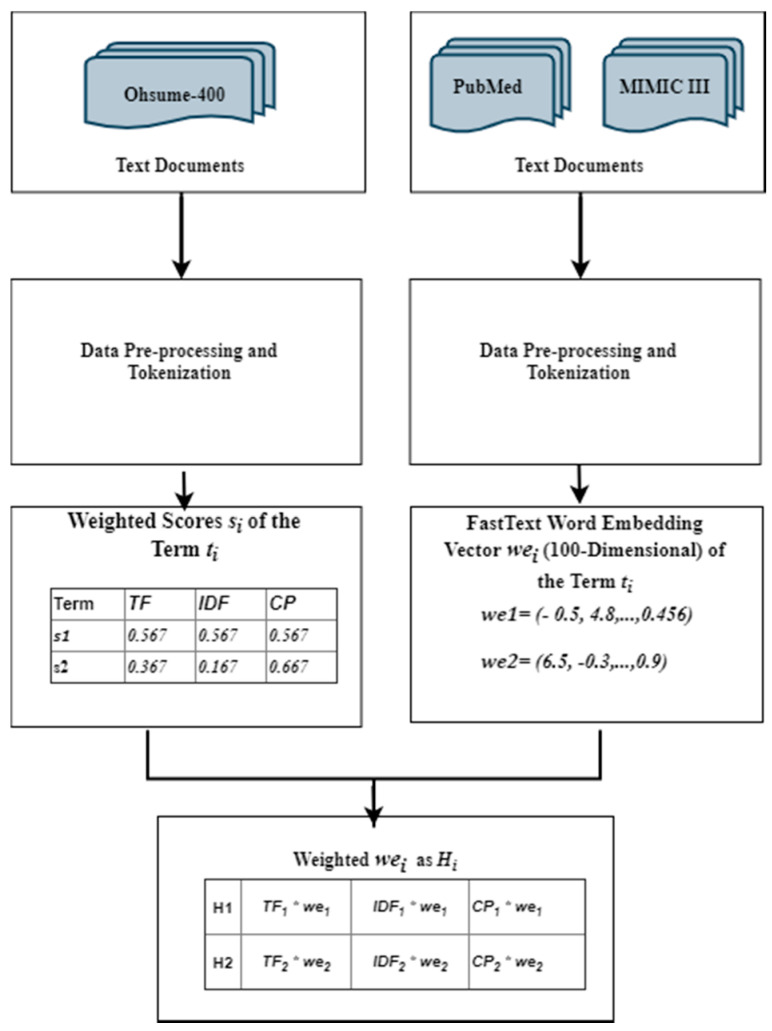
The steps of the weighted feature representation.

Figure 1 shows how the combination process is done in detail. 

### 3.4. Multiclass Classification Model

**Figure 2:**
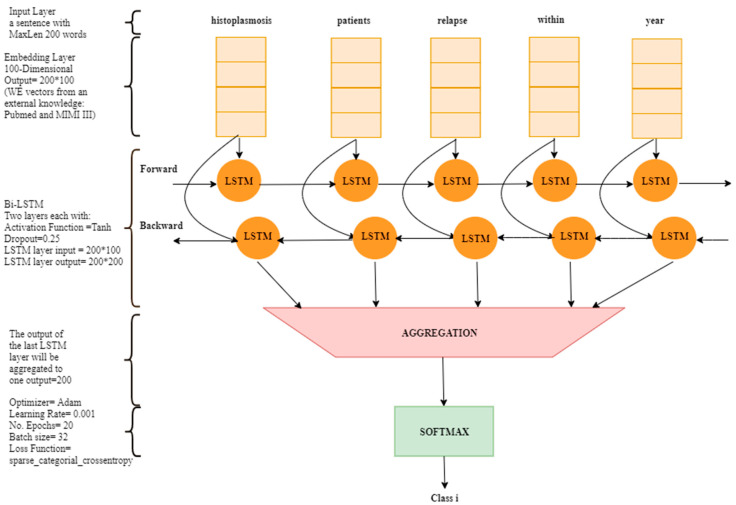
The architecture of our bidirectional long short-term memory (BLSTM) multiclass classification system.

After obtaining the combined vector of features, we needed to test it in a case study that is represented by a multiclass classification problem. For this we used the OHSUMED dataset [25] and the vector of features as an input, then one output over 23 classes was predicted on each test data collection. First, we created a multiclass classification model depending on a bidirectional long short time memory (BLSTM). The BLSTM model has the ability to capture patterns and parse sentences in both directions. However, this model takes a longer learning time in comparison to a simple LSTM or Recurrent Neural Network (RNN). This model consisted of the following layers, where Figure 2 depicts them more clearly:

The input layer: this layer takes each document (i.e., a sequence of words) as input to the neural network.

The embedding layer: this layer maps between each word in the input text with the embedding list. At that time, it retrieves a floating point vector for each word. However, the floating-point value can be created from the training dataset or from different pretrained external knowledge. Thus, we used external knowledge that was extracted from PubMed and MIMIC III datasets. Hence, our input data are represented as vectors of features.

Bidirectional LSTM layers: we created one BLSTM layer which consists of the hyperparameters of Tanh for activation function and a dropout value of 0.25.

The output layer: this layer is responsible for predicting the output class. Since this model is a multiclass model, then it must predict one of the 23 cardiovascular diseases’ categories. We selected SoftMax as an activation function in this layer because it is suitable in a multiclass problem.

Additionally, we empirically optimized some important hyperparameters that eased the learning process and gave good results. We chose the Adam optimizer and set learning rate to 0.001 and decay value to 1 × 10^−6^, sparse_categorical_crossentropy loss function with 20 epochs and for a batch size of 32.

## 4. Experimentation and Results

To evaluate and compare our proposed methods, we conducted a set of experiments and analyzed the performance results of the deep learning architecture of BLSTM with other architecture and machine learning approaches. In this section, we also present all the performance measurements used in our model assessment.

### 4.1. Experimentation Setup

We encountered several problems when we tried to deal with PubMed and MIMIC-III datasets. Our limited RAM and processor resources could not manage the large size of the data. We used two main hardware resources to deal with this problem, namely Google Colab and TRUBA infrastructures. We first experienced the use of Google Colab. Its tensor processing unit (TPU) consists of more than 100 GB RAM and 30 GB of disk space. This infrastructure worked well for conducting experiments to evaluate our model but, unfortunately, it was not able to complete the task when we attempted to use the same environment to create the FastText WE vector. Instead, we opted for TRUBA, which is a computer cluster system provided by Turkish National Science Foundation (TÜBİTAK) and includes high-performance computing hardware and data storage resources. We used a server consisting of 128 nodes, each with eight cores of two Xeon E5-2690 2.90 GHz CPUs and 256 GB RAM. For the software stack we used Python 3.6, as well as Keras and TensorFlow libraries, for creating our neural network. We also used the Gensim Python library to create both the FastText WE vector and the weighted scores.

The primary experiments were performed only for setting up and fixing hyperparameter values of our BLSTM model. We tried several hyperparameters, such as dropout values, between 0.1 and 0.5, then we explored more activation functions, namely Tanh, Sigmoid and Relu. After that, we tried some epochs values, between 10 and 32, and several batch-size values between 10 and 32. Finally, we tried some optimizers such as ADAM, SGD, and RMSprop. As a result, we fixed all the BLSTM hyperparameters as mentioned in Section 3.3.

We performed all experiments for multiclass classification of biomedical text using the OHSUMED-400 dataset, which contains a total of 9200 text documents. We went through the dataset and recognized that some documents had more than one class, and since we were working with a multiclass problem we removed all the redundant documents. Hence, the remaining number of documents became 7512 documents. We divided the remaining documents into two parts: 80% as training (6010 documents) and 20% as testing (1502 documents). As a result, each document was labeled with only one class over 23 cardiovascular disease classes.

The first experiment was performed on the original WE vector (i.e., the WE before the combination operation) as a baseline. We did it to observe how the weighted feature representation systems could affect the performance of the classification model in comparison to the original WE. After that, we applied the same BLSTM environment and hyperparameters to the other hybrid feature vectors: HTF, HIDF and HCP.

We also performed some extra experiments on some state-of-the-art machine learning algorithms and approaches. We tried our originally created feature vectors at this stage. Accordingly, we chose to work with a set of ML algorithms, namely logistic regression (LR), *k*-nearest neighbors (*k*-NN) (with *k* = 5), random forest (RF), support vector machine (SVM), linear support vector machine (LSVM), decision tree (DT), multilayer perceptron (MLP) and Gaussian naïve based (GNB) classification. In these experiments, we considered the default settings of their hyperparameters.

We also compared our models with one of the closest related studies from the literature. In that study [2], they tested their created word-embedding based features representation approach on the same OSUMED-400 data that we used in this study. In fact, we assessed our model using the same performance measurements that were mentioned in their study. Although they proposed similar word-embedding based modeling, it was not a specifically trained model for biomedical domain. Using the same dataset as their study allowed us to use it for comparison purposes.

### 4.2. Evaluation Metrics

We compared our performance results in terms of two commonly used measurement metrics, namely training accuracy and validation accuracy. These measurements are well-known in many neural network applications.

Accuracy is the ability of our model to classify documents correctly. However, there are two popular types of accuracy in neural networks, namely training accuracy and validation accuracy [26]. Training accuracy is the capability of our model to classify the data correctly during the training process. Validation accuracy shows how good and accurate our model is during the testing and validation process. Both are calculated in each epoch of the neural network.

On the other hand, loss metric computes the error in the prediction of the classification model that can occur during fine-tuning the weights of the neural network [26]. Hence, there are two types of this metric that are related to the training and validation processes.

### 4.3. Experimentation Results

In this part we present all the results we obtained from experimentation mentioned in the previous subsection. We discuss all the results in terms of training and validation accuracies and loss. Furthermore, we also present the results of our BLSTM model with both the original baseline FastText WE system and the weighted feature representation techniques using the aforementioned metrics. We also present the results of the experiments to provide a comparison with other commonly used ML algorithms for related multiclass research.

#### 4.3.1. BLSTM Multiclass Classifier Results

According to the validation accuracy and loss in Table 2, the highest performance was accomplished by the CP weighting technique by obtaining 0.494 and 1.901, respectively. We also recognize that there was only a 0.01 difference in validation accuracy between CP and the other weighting techniques, i.e., TF and IDF. Alternatively, the baseline results showed the best training accuracy and loss amongst them all. In the end, all the weighting techniques showed their best performance in comparison with the baseline in terms of validation accuracy.

#### 4.3.2. Results of Other ML Approaches

We notice from Table 3 that the second-best performance among ML algorithm was the linear SVM in terms of accuracy score. On the other hand, decision trees didn’t respond very well to the original FastText WE system. Generally, the original FastText WE technique produced the highest score with our BLSTM model in comparison with the other ML algorithm.

### 4.4. Discussions and Comparisons of the Results

In the last section, the CP weighting technique gave the highest scores amongst all the feature weighting techniques when they were used in the BLSTM model. However, there was only a small difference, about 1%, between CP and the two other weighting techniques. Moreover, all the weighting techniques also registered higher performance scores in comparison to the baseline experiment. To explain why these weighting methods succeeded, we must look deeply into the work principle of the WE systems. WE tends to create a feature vector of a character, word, sentence or even an entire document. It is able to discover syntax and context relations between words [1,2]. Each number in the vector gives a meaningful explanation about the word it describes. Therefore, in our opinion, changing this representation may affect the performance of the classification system positively or negatively. In our case, the weighting methods operated positively by adding the term statistical information, namely TF, IDF and CP, into the WE vector. Moreover, CP registered the highest performance, which explains how the class information can contribute to enhancing accuracy.

During experimentation of the baseline model, we noticed that assigning the parameter Trainable = True or False in the embedding layer, was an important factor in enhancing or degrading the performance of the neural network. Assigning this parameter to True would use the training data, which in our case was the OHSUMED dataset, to create the embedding vectors. Thus, the OHSUMED documents number was not sufficient for creating vectors and could lead to a server over-fitting problem. Additionally, if we used this parameter equal to True with our external knowledge, the structure of the vectors obtained from that external dataset would be changed and degrade the accuracy of the model. Therefore, we assigned this parameter to False in our case with the baseline experiments. This means using external knowledge had the advantage of enriching and increasing the learning ability of the neural network.

We tried to control overfitting problems as much as we could by adding some regularizations into our model such as dropout hyperparameter and shuffling the dataset before splitting. For that reason, the training accuracy of the baseline was higher than the other weighting techniques, i.e., about 0.961. Accordingly, we chose to rely on validation accuracy to evaluate our model’s performance.

Label dependency plays an important role to enhance or degrade the total performance of any classification system. In a multilabel classification problem, this phenomenon behaves positively to enhance the total performance of the system [27,28,29]. In this case, one needs to increase the probability that multiple classes can exist together in a particular document, while label dependency is considered a curse for some multiclass problems such as the OHSUMED-400 dataset [3]. Each document in the OHSUMED-400 dataset has only one related class. Besides, this data describes 23 types of cardiovascular diseases, i.e., describing 23 different but related diseases and conditions of heart and blood vessels. This makes the classification process more complicated to identify unique patterns of each class, which means that classes can share features since they share the same disease conditions. Hence, sharing features between classes means that they are dependent and closed to each other. Consequently, the general performance in all cases regarding the OHSUMED dataset was low, i.e., they didn’t get improved to higher scores. The large number of the classes in the classification process had a negative effect in our model. Because the more the number of classes involved in the classification process, the harder it was to identify each class correctly.

**Figure 3:**
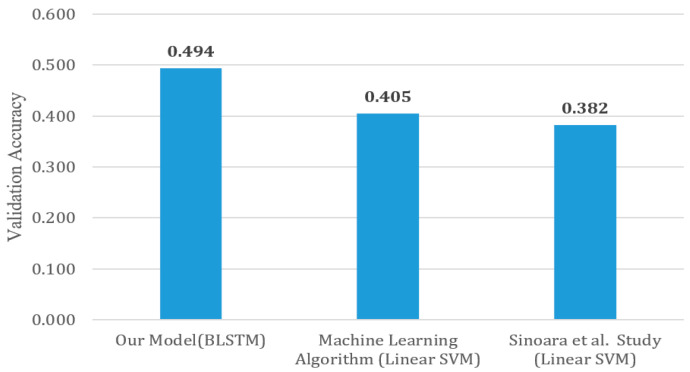
A comparison between the best scores of deep neural networks with BLSTM architecture, other common ML algorithms and a literature study.

We also compared our results to very similar research in the literature dealing with creating a customized WE system and applying it on the OHSUMED-400 multiclass dataset. To that purpose, Sinoara et al. [2] tried a WE model with several ML models, namely linear SVM, NB, decision tree and k-NN, where each model attained 0.3821, 0.279, 0.101 and 0.3076 in terms of validation accuracies, respectively. Hence, our model outperformed their scores using BLSTM and linear SVM algorithms. Figure 3 shows a chart that compares the best scores of BLSTM and ML and the best score from the study of Sinoara et al. [2].

## 5. Conclusions

The multiclassification problem aims to identify one and only one class over multiple classes for a particular document. This presents us with a serious challenge in that we must discover the unique features and patterns for each class individually. Therefore, if there are associations or relationships between classes (i.e., label dependency) this may negatively impact the multiclass classification of documents.

Our aim in this study was to identify unique features and patterns on the OHSUMED-400 multiclass dataset, which is a biomedical text document collection for cardiovascular diseases. This could be accomplished by enhancing the feature-representation technique used as a primary stage during the classification process. Hence, we created a weighted feature-presentation technique that combined the advantages of both, i.e., word embeddings (WE) and bag-of-words (BoW) techniques. We created WE FastText vectors from two datasets: Pubmed and MIMIC III. Then, we used the OHSUMED-400 dataset to create three main statistical weighting scores for each term in the documents such as term frequency (TF), inverse document frequency (IDF) and class probability (CP). After that, we tested our weighted feature of vectors on a case study which was represented as a multiclass problem, and used it to predict one of the 23 cardiovascular disease categories.

The results showed that our classification model, which depends on bidirectional LSTM and CP weighted feature-representation technique, registered the highest performance score. Moreover, it recorded higher results even when compared to some machine learning (ML) algorithms and one literature study. It seems that class-term relationship information gives a positive impact to improve the performance of the multiclass classification system.

We discovered that by adding useful information and weights to the original WE vector, our classification model worked better. Furthermore, we found that the general performance of classifying the OHSUMED dataset was low because it describes multiple intervolved and related diseases and conditions of the heart and blood vessels. Additionally, the OHSUMED dataset includes 23 classes, which makes the classification process more complex. Hence, the performance degrades because of large number of classes in the process.

In the future, we are planning to take our work forward to a semantic level. We will try to include different external biomedical knowledge resources, such as UMLS dictionaries, to semantically extend our weighted feature representation model. Additionally, we will try to extract biomedical named entities from the dataset, which are represented as treatments, procedures and diseases. We hope that this will help us to explain our model behavior accurately.

## Figures and Tables

**Table 1:** Basic statistics about the three datasets used in this study.

Descriptions	PubMed	MIMIC III	OHSUMED-400
Total number of documents	2,000,000	2,083,180	9200
Total number of sentences	13,218,036	40,378,672	76,863
Total number of tokens	300,577,868	606,768,871	1,658,785
Total number of characters	1,959,234,200	3,458,104,347	10,797,237
Max document size in sentence	62	559	23
Max document size in tokens	1624	8770	605
Max document size in characters	9954	55,293	4025
Average document size in sentence	6	19	2
Average document size in token	150	291	180
Average document size in characters	980	1660	1173
Total number of classes	N/A	N/A	23

**Table 2:** Training and validation accuracy and loss results of the BLSTM model with all the proposed feature vector techniques (bold scores show the best result in a row).

Metrics	Baseline FastText WE	TF Weighted	IDF Weighted	CP Weighted
Training Accuracy	**0.962**	0.690	0.724	0.680
Validation Accuracy	0.454	0.486	0.483	**0.494**
Training Loss	**0.149**	1.336	0.899	1.036
Validation Loss	3.011	1.966	2.018	**1.901**

**Table 3:** The performance of our original FastText WE system that was used in some ML algorithms in terms of validation accuracy (bold indicates best scores of all studies).

Machine Learning Approach	Accuracy
Deep Neural Network (DNN) with BLSTM	**0.454**
Logistic Regression	0.377
k-NN (k = 5)	0.279
Random Forest	0.271
Multilayer Perceptron	0.321
Gaussian Naive Bayes	0.309
SVM	0.383
SVM (Linear kernel)	0.405
Decision Tree	0.124
